# A Deep Metagenomic Snapshot as a Proof‐of‐Concept for Resource Generation: Simultaneous Assembly of Host, Food, and Microbiome Genomes From Stingless Bee Larval Food

**DOI:** 10.1002/ece3.72546

**Published:** 2025-11-29

**Authors:** Carlos Ueira‐Vieira, Ana Carolina Costa Santos, Thayane Nogueira Araújo, Solange Cristina Augusto, Natanael Borges de Avila, Ana Maria Bonetti, Anderson Rodrigues dos Santos

**Affiliations:** ^1^ Laboratory of Genetics, Institute of Biotechnology Federal University of Uberlândia Uberlândia Brazil; ^2^ Laboratory of Bee Ecology, Institute of Biological Sciences Federal University of Uberlândia Uberlândia Brazil; ^3^ Faculty of Computer Science of Federal University of Uberlândia Minas Gerais Brazil

**Keywords:** genome assembly, genomic resources, NGS, pollinator, proof‐of‐concept, stingless bee

## Abstract

Characterizing the complex web of ecological interactions is a central challenge in molecular ecology. Shotgun metagenomics of environmental samples offers a powerful, high‐resolution approach, yet its potential for simultaneously generating multiple genomic resources from different trophic levels remains underexplored. This study serves as a proof‐of‐concept, using deep sequencing of a single, complex sample—the larval food of the stingless bee *Tetragonisca angustula* —to demonstrate the method's capacity to recover genomic information across varying template abundances. We successfully assembled three genomes of different completeness levels: a near‐complete bacterial genome (*Acetilactobacillus jinshanensis*, 2,097,977 bp with 0.002% ambiguous bases), a draft mitochondrial genome ( *T. angustula* , 15,498–15,549 bp), and a fragmented chloroplast genome ( *Lactuca sativa* , 130,532 bp with 23.47% ambiguous bases). The assembly quality gradient, observed from complete to fragmented, directly reflects the relative abundance of each DNA template in the environmental sample, demonstrating the method's sensitivity and ecological informativeness. Beyond these genomic resources, the data provided a comprehensive biodiversity profile, revealing DNA from seven major taxonomic groups, including 209 bacterial genera, 123 plant families, and 55 insect taxa. Additionally, genomic comparisons using Average Nucleotide Identity (ANI) and digital DNA–DNA Hybridization (dDDH) analyses suggest that the dominant bacterial strain represents a putative novel species within the genus *Acetilactobacillus*. This approach simultaneously provided insights into host genetics, food sources, and microbial communities, illustrating the potential of single metagenomic datasets to generate multiple valuable genomic resources for molecular ecology research.

## Graphical abstract

This study serves as a proof‐of‐concept, demonstrating that deep shotgun sequencing of a single complex sample—stingless bee larval food—can simultaneously generate multiple genomic resources. From this dataset, we assembled three genomes (a near‐complete bacterium, a draft host mitochondrion, and a fragmented plant chloroplast) with varying completeness. This assembly quality gradient directly reflects the relative abundance of each DNA template, showcasing the method's sensitivity and its utility for providing ecological insights from genomic data.

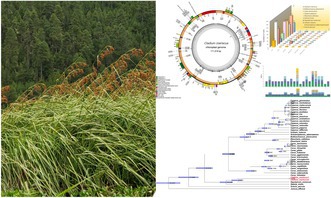

## 1 Introduction

The stingless bee *Tetragonisca angustula* , commonly known as ‘Jataí’ in Brazil, is widely distributed across tropical and subtropical regions of Central and South America. Renowned for its remarkable adaptability, this species thrives in diverse environments, including those heavily influenced by human activities. Its ability to exploit various nesting sites and floral resources within anthropized areas contributes to its ecological success (Fierro et al. 2012; de Novais et al. 2014; Prado et al. 2021).

Beyond its wide distribution and adaptability, *T. angustula* bees exhibit a highly organized colony structure typical of social insects. Their nests are often constructed in hollow trees, wall cavities, and other sheltered locations, utilizing materials such as resin, mud, and plant fibers (de Novais et al. 2015; Prado et al. 2021; Silva et al. 2021). These bees are integral to meliponiculture—the practice of rearing stingless bees for their products, including honey, geopropolis, and wax (Pucciarelli et al. 2014; Santos et al. 2023; Alves et al. 2024). *Tetragonisca angustula* honey is particularly valued for its unique flavor and medicinal properties (Torres et al. 2018; Valcanaia et al. 2022; Schloss et al. 2011; Vit et al. 2018, 2024).

The larval food of *T. angustula* consists of a mix of fermented pollen and nectar, as well as glandular secretions, providing essential nutrients for the growing larvae (Hartfelder and Engels 1989; Kraus et al. 2020). This nutrient‐rich substance supplies essential macronutrients and harbors a diverse microbiota, crucial for food fermentation, pathogen defense, and overall larval health. Studies have shown that this microbiota is diverse, including various bacterial and fungal species that contribute to the overall health and development of the larvae (Menezes et al. 2013; Menezes et al. 2015; de Paula et al. 2021; Santos et al. 2023).

Molecular techniques are widely used in microbial ecology studies to gain a deeper understanding of the composition and function of these microbial communities. Specifically, sequencing of the 16S rRNA and Internal Transcribed Spacer (ITS) regions has become the standard approach for profiling bacterial and fungal communities. The 16S rRNA sequencing is specifically used for identifying and classifying bacteria and archaea based on the highly conserved 16S ribosomal RNA gene (Abellan‐Schneyder et al. 2021). ITS (Internal Transcribed Spacer) sequencing, on the other hand, targets fungal communities by focusing on the ITS region, which is a non‐coding area between the small‐subunit rRNA and large‐subunit rRNA genes (Ceballos‐Escalera et al. 2022). Both the 16S and ITS regions are widely used for fingerprinting microbial communities because they contain variable sequences that can be used to distinguish between different species or strains. Still, they provide information primarily on taxonomic composition, with limited insight into the full genomic content of these communities (Ceballos‐Escalera et al. 2022; Matchado et al. 2024). Recently, our research group applied Next‐Generation Sequencing (NGS) techniques to analyze the 16S and ITS regions of larval food from four species of Brazilian stingless bees (Santos et al. 2023). These efforts contribute to the expansion of genomic databases and enhance our understanding of the microbiomes associated with native pollinators, which are crucial for maintaining ecosystem stability.

Despite the effectiveness of 16S and ITS sequencing in taxonomic identification, these targeted techniques do not capture the full genetic diversity within a sample. To achieve a more comprehensive view, NGS techniques offer alternative approaches, such as metagenomic shotgun sequencing. Metagenomic shotgun sequencing involves sequencing all the genetic material from a sample, which includes DNA from all organisms present (Ranjan et al. 2016). This technique does not target specific genes or regions but rather captures the entire genomic content of the communities in the environment. It allows for a high‐resolution view of taxonomic diversity, often down to the strain level, and enables the recovery of complete genomes from the most abundant organisms (Zhang et al. 2021).

This study serves as a high‐resolution proof‐of‐concept, demonstrating the power of deep shotgun sequencing of a single, complex environmental sample to generate multiple genomic resources simultaneously. We employed this approach to perform a deep analysis of DNA extracted from the whole larval food of a single *T. angustula* colony in an urban setting. We showcase its utility not only for deep biodiversity profiling but also for the simultaneous generation of key genomic resources, including the host's mitochondrial genome, the chloroplast genome from a key plant resource, and the complete genome of a novel, predominant bacterial species. This high‐resolution snapshot showcases the potential of shotgun sequencing as a powerful tool for uncovering intricate biological interactions and identifying novel genomic components. This framework not only uncovers intricate biological interactions and identifies novel genomic components, but can also lead to the discovery of new taxa.

## 2 Material and Methods

### 2.1 Sample

The larval food (LF) of *T. angustula* was the same as that used in our previous work (Santos et al. 2023) from an urban meliponary in Uberlândia city, Minas Gerais, Brazil. The sample was collected from a single disc of brood cells in a beehive in October 2018.

Brood cells were collected, placed in sterile Petri dishes, and transported to the laboratory for further processing. Within a laminar flow cabinet, the brood cells were cleaned with sterilized distilled water for 1 min and then rinsed three times with 70% ethanol. Afterward, the brood cells were carefully opened using a sterile pipette tip, and the eggs were removed. The LF was then collected, ensuring that the LF from larval cells was discarded.

### 2.2 DNA Extraction and Shotgun Sequencing

A 100 μL of whole larval food (LF) was used for DNA extraction. The procedure followed the manufacturer's instructions for bacterial and fungal DNA extraction using the DNeasy Blood & Tissue Kit (Qiagen). DNA quantity was measured using a NanoDrop 2000 Spectrophotometer, and its quality was assessed by electrophoresis on a 1% agarose gel stained with ethidium bromide.

Subsequently, 1000 ng of DNA was used to construct the whole‐genome library, following the BGI Americas in‐house protocol. Library quality was assessed using the Agilent 2100 Bioanalyzer (Agilent Technologies, Santa Clara, CA, USA), and paired‐end (150 bp) sequencing was performed using the DNBseq sequencing strategy.

### 2.3 Scaffolding Assembly

Raw data containing adapter sequences or low‐quality reads were filtered. Initially, data processing was undertaken to remove contaminants and obtain valid data using SOAPnuke software (version 2.1.7) with an output read quality value system set to Phred+33. Afterward, the scaffolds were assembled using the SPAdes genome assembler version 3.14.0, employing the default k‐mer values (21, 33, 55, and 77 bp) (Santos et al. 2023).

### 2.4 Shotgun Metagenomic Diversity Analysis

The scaffolds were subjected to BLASTN searches against the entire offline NCBI database (downloaded in March 2023) for the diversity analysis. E‐value threshold of 10^−10^, and only the best top hits were used in diversity analysis.

### 2.5 *T. angustula* Mitochondrial and Plant Chloroplast Assembly and Annotation

To reconstruct the organellar genomes, two complementary assembly strategies were employed. The first assembly was a *de novo* approach using GetOrganelle (version 1.7.6) directly from the raw reads. The second was a reference‐guided scaffolding approach using RagTag (version 2.1), with the mitochondrial genome (NCBI ID OR030859.2) or chloroplast genome (NCBI ID AP007232.1) as a reference and the scaffolds from SPAdes assembled. As *Lactuca sativa* was identified as the most representative plant in our initial BLAST analysis, its chloroplast was selected for targeted assembly. Both assemblies were conducted on the Galaxy platform (usegalaxy.eu).

The mitogenome was annotated using Annotator for Genome of Organelle from Referenced sequence Analysis (AGORA) online access Jung et al. (2018), and the chloroplast genome was annotated using the GeSeq Annotation of Organellar Genomes (Tillich et al. 2017).

### 2.6 Bacterial Genome Assembly and Annotation

A distinct, custom reference‐guided pipeline was used for assembling the most abundant bacterial genome. First, as *Acetilactobacillus jinshanensis* was identified as the most abundant species based on our initial BLAST analysis (see below), its genome was selected for targeted assembly. We selected the reference genome for assembly by analyzing all BLASTn results of all scaffolds against all NCBI nucleotide sequences, running the analysis offline on the laboratory server. The approach prioritizes alignment quality with NCBI sequences and then evaluates the representativeness of each match (i.e., each NCBI subject) within the overall context. To achieve this, the alignment identity is multiplied by the alignment length for each match. For each NCBI subject, the products of identity and alignment length from all its matches are accumulated (summed). Finally, each subject's accumulated value is divided by the total accumulated value from all matches. This normalization yields a percentage of representativeness for the genome of each species. The sum of all values is equal to 1, representing a probability distribution across species.

Subsequently, we assembled the *Acetilactobacillus jinshanensis* strain ajita genome, using the genome GCA_004359375.1 as a reference. The reference sequence served as a scaffold to align sequencing reads. The information from these aligned reads was then used to generate an improved consensus sequence, effectively refining or replacing the initial reference based on the experimental data.

The process commenced by indexing the reference DNA sequence using SMALT (version 0.7.6). Subsequently, SMALT aligned sequencing reads (paired‐end data) against this indexed reference. The resulting alignments underwent standard processing using SAMtools (version 1.17), including format conversion, filtering to retain mapped reads, coordinate sorting, and indexing for efficient data retrieval. Next, bcftools (version 1.17) mpileup was employed on the processed alignments to determine the consensus base call at each position covered by the reads, relative to the original reference coordinates. Based on this pileup information, a new consensus sequence was constructed by selecting the most probable base at each position derived from the aligned sequencing data. This sequence underwent necessary post‐processing steps and was formatted into the standard FASTA format. Finally, this newly generated consensus sequence, derived from the empirical sequencing data aligned to the initial reference, was used to create our *Acetilactobacillus jinshanensis* strain ajita genome.

For functional annotation, the genome annotation pipeline PANNOTATOR (version 1.0) was employed. The genome was analyzed using ABRicate (version 1.0.1) on the Galaxy platform to screen for antimicrobial resistance and virulence genes. The antiSMASH 7.1.0 software was used to predict gene clusters responsible for the biosynthesis of secondary metabolites.

### 2.7 Genomic Comparison and Species Delineation

To assess the taxonomic novelty of the *Acetilactobacillus jinshanensis* strain ajita, we performed whole‐genome comparisons using Average Nucleotide Identity (ANI) and in silico DNA–DNA Hybridization (dDDH). ANI values were calculated using FastANI (version X.X) against the reference genome of *A. jinshanensis* (GCA_004359375.1) and other publicly available *Lactobacillaceae* genomes. The dDDH values were obtained using the Genome‐to‐Genome Distance Calculator (GGDC 3.0) web server, employing its recommended Formula 2. Established thresholds for species delineation of < 95%–96% for ANI and < 70% for dDDH were used to interpret the results. The genomes used for comparison included: *Lactobacillus amylovorus* (CP002609.1), *Lactobacillus delbrueckii* (CP018218.1), *Lactobacillus apis* (CP029476.1), *Lactobacillus amylolyticus* (CP031835.1), *Lactobacillus intestinalis* (CP072983.1), and *Lactobacillus acidophilus* (CP139575.1).

### 2.8 Palynological Analysis

The pollen sample underwent acetolysis according to Erdtman's (1960) methodology. It was identified using references from Labouriau and Joly (1971) and an image collection (Funed‐Pol) available in the Species Link network (speciesLink network 2025). Additionally, pollen from plant specimens collected in the study sites was used for comparison. The samples were examined under a Carl Zeiss Axio Imager.M2 microscope to analyze pollen composition. Identification and quantification were carried out by preparing two permanent slides, with each coverslip divided into four quadrants. One hundred pollen grains per quadrant were counted, summing up to 800 grains per sample. The number of distinct pollen types present determined pollen richness. At the same time, diversity, representing the food niche breadth, was evaluated using the Shannon‐Wiener index (H′), as described by Camillo and Garofalo (1989).

## 3 Results

### 3.1 DNA Sequencing and Biodiversity Analysis

A total of 200,260,567 reads and 60,078,170,100 bases, with a GC content of 40.44%, were obtained after sequencing and trimming (Phred +33). The assembly yielded 64,212 scaffolds, ranging from a minimum length of 56 bp to a maximum length of 823,205 bp, with a median length of 653 bp.

**FIGURE 1:**
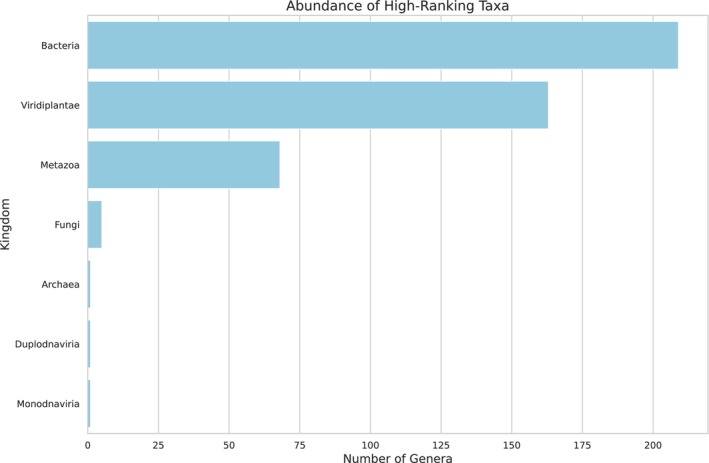
Abundance of high‐ranking taxa in the larval food of *Tetragonisca angustula* based on shotgun metagenomic sequencing data.

The BLASTn analysis demonstrated that the scaffolds aligned with the offline NCBI database, enabling the investigation of the sample's biodiversity. Only 12.61% of scaffolds had matches, demonstrating the enormous DNA diversity in nature, which contrasts with what is already known in the NCBI dataset. Seven different high‐ranking taxa were identified (Figure 1).

**FIGURE 2:**
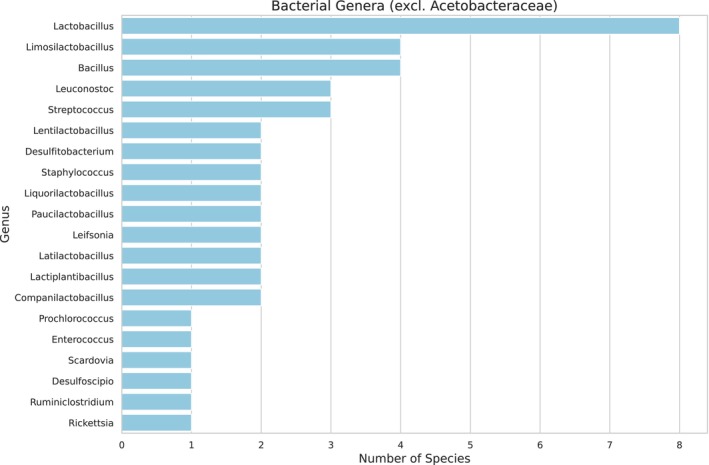
Abundance of bacterial genera by family in the larval food of *Tetragonisca angustula* , based on shotgun sequencing analysis. The Acetobacteriaceae family is excluded due to its predominance, but other genera from different families are highlighted.

**FIGURE 3:**
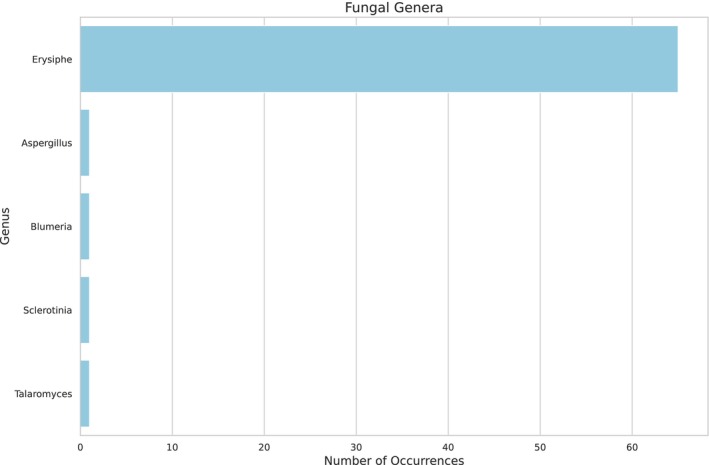
Abundance of fungal genera by family in the larval food of *Tetragonisca angustula* , based on shotgun sequencing. The distribution of fungal families highlights the diversity within the larval food community.

**FIGURE 4:**
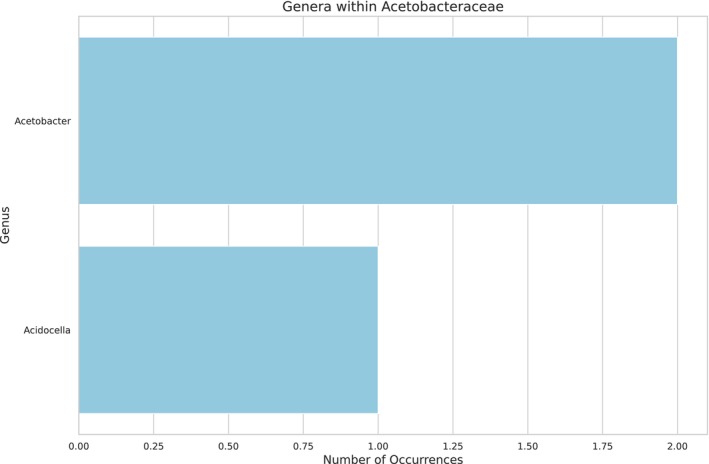
Abundance of bacterial genera within the family Acetobacteriaceae in larval food of *Tetragonisca angustula* based on shotgun sequencing. The top genera with the highest abundance are represented, highlighting their prevalence in the larval food community.

As the larval food consists of fermented pollen and nectar, a high diversity of microorganisms and plants is expected. Two hundred nine bacterial genera (Figure 2, see Appendix A Table A1) and five fungal genera were identified (Figure 3, see Appendix B Table B1). The most abundant bacterial family was Acetobacteriaceae (Figure 4), which includes the genera *Acetolactobacillus* and *Lactobacillus*, which were the most prevalent in the sample.

**FIGURE 5:**
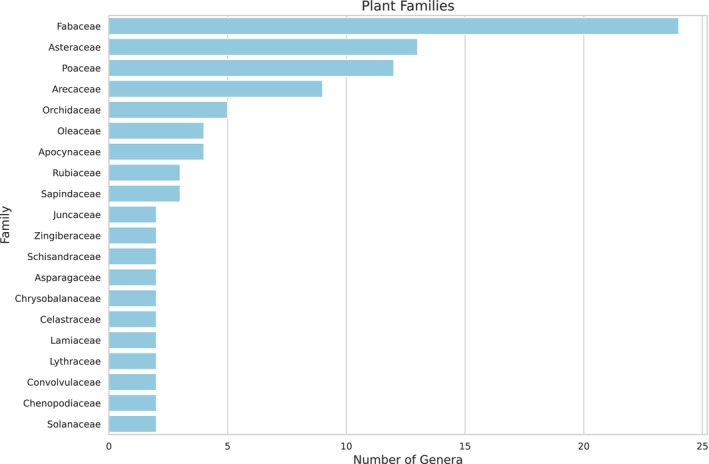
Based on shotgun sequencing, the most abundant family of plants in the larval food of *Tetragonisca angustula is illustrated*. The distribution of plant families highlights the diversity.

A total of 123 plant families and 166 genera were identified in the larval food of *T. angustula* , with the most abundant families represented in Figure 5 (see Appendix C Table C1).

In the palynological analysis conducted through microscopy, a total of 27 pollen types were identified, belonging to 14 botanical families: Amaranthaceae, Apocynaceae, Arecaceae, Asteraceae, Brassicaceae, Celastraceae, Commelinaceae, Fabaceae, Lamiaceae, Malpighiaceae, Malvaceae, Myrtaceae, Solanaceae, and Vochysiaceae. Additionally, six pollen types remained unidentified (Table 1). Among the identified families, Solanaceae was the most abundant, comprising 24.97% of the total pollen count, followed by Asteraceae (Ra = 21.72%) and Fabaceae (Ra = 8.24%).

**TABLE 1:** Relative abundance (Ra) of pollen types used by *Tetragonisca angustula* .

Family	Species	Ra (%)
Amaranthaceae	*Alternanthera brasiliana*	0.62
	*Amaranthus* type	0.75
Apocynaceae	*Mandevilla* type	5.49
Arecaceae	*Syagrus romanzoffiana*	7.62
Asteraceae	*Baccharis* type	3.87
	*Lepidaploa* type	9.49
	*Tridax* type	8.36
Brassicaceae	*Artemisia* type	3.50
Celastraceae	*Maytenus ilicifolia*	1.12
Commelinaceae	*Commelina benghalensis*	2.75
Fabaceae	*Cenostigma pluviosum*	6.62
	*Delonix regia*	0.12
	*Mimosa* type	0.12
	*Myroxylon peruiferum*	1.37
Lamiaceae	*Hyptis* type	0.20
Malpighiaceae	*Banisteriopsis* type	0.50
	*Malpighia emarginata*	1.00
Malvaceae	*Eriotheca* type	0.25
Myrtaceae	*Eucalyptus* type	7.62
Solanaceae	*Solanum* type	24.97
Vochysiaceae	*Vochysia* type	0.12
NI	Sp 1	6.49
	Sp 2	2.75
	Sp 3	3.00
	Sp 4	0.62
	Sp 5	0.50
	Sp 6	0.25
Shannon‐Wiener Index (H′)	H′	2.603
Pielou's evenness Index	J	0.789
Richness		27

Abbreviation: NI, pollen types not identified.

**FIGURE 6:**
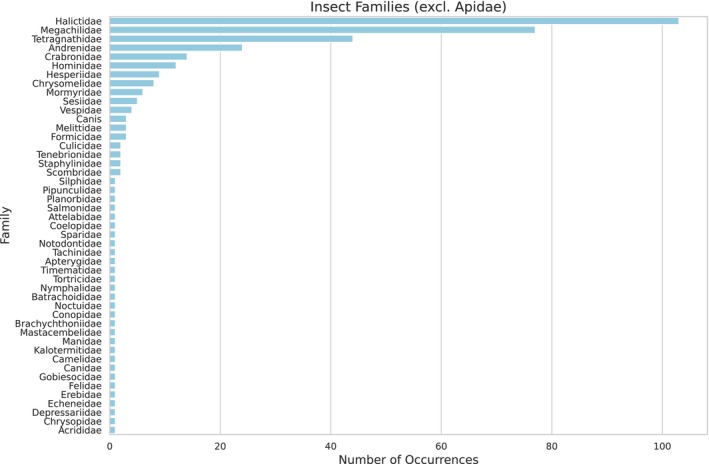
An abundance of insect families (except Apidae) is present in the larval food of *Tetragonisca angustula* . Data were obtained through shotgun sequencing analysis, highlighting the diversity of insects associated with the hive environment.

**FIGURE 7:**
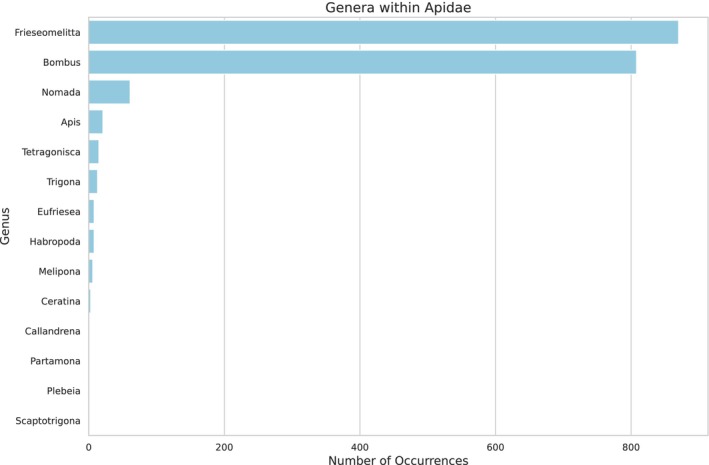
Based on shotgun sequencing analysis, genera within the family Apidae were identified in the larval food of *Tetragonisca angustula* . The figure highlights the diversity of bees associated with the hive environment.

A total of 55 insect taxa were identified in the larval food of *T. angustula* (see Appendix D Table D1), with the most abundant families represented in Figure 6. The family Apidae was excluded from the graph due to its overwhelmingly high abundance, which could distort the visual representation of the relative proportions of the other insect families. The most predominant genera of Apidae were *Frieseomellita* and *Bombus* (Figure 7).

### 3.2 *T. angustula* Mitochondrial Genome Assembly

The output from the GetOrganelle assembly (ab initio) yielded a mitochondrial genome of 15,498 bp, whereas the RagTag assembly produced a genome of 15,549 bp. Both assemblies represent draft‐quality mitochondrial genomes that successfully recovered the core protein‐coding genes essential for mitochondrial function. The assemblies contained 10 protein‐coding genes (nad1, nad2, nad4, nad5, nad6, cox1, cox2, cox3, atp6, and cytb), representing the majority of genes involved in oxidative phosphorylation and electron transport.

**FIGURE 8:**
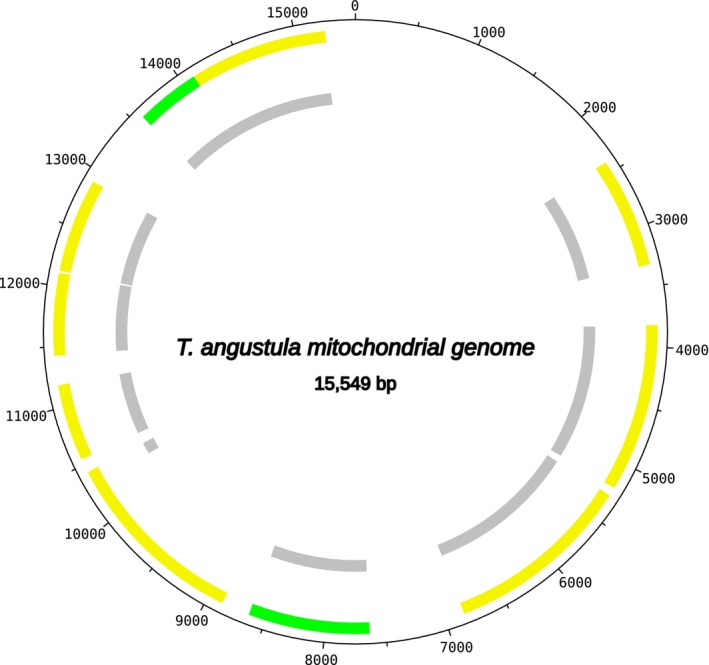
Schematic representation of the mitochondrial genome of the *Tetragonisca angustula* assembled in this study.

However, the assemblies lacked several essential mitochondrial components typically found in complete insect mitogenomes, including: (i) ribosomal RNA genes (rrnL and rrnS), which are critical for mitochondrial protein synthesis; (ii) the nad3, nad4l, and atp8 genes; and (iii) the complete set of 22 tRNA genes necessary for mitochondrial translation. The absence of these genes indicates that while the assemblies captured the core metabolic machinery, they represent partial recoveries of the complete mitochondrial genome, likely due to the moderate abundance of mitochondrial DNA relative to other templates in the metagenomic sample (Figure 8).

### 3.3 Plant Chloroplast Genome Assembly

The chloroplast genome of *Lactuca sativa* , the second most representative plant in the pollen sample, was assembled with a total length of 130,532 base pairs (bp). However, this assembly represents a highly fragmented, draft‐quality genome containing 30,641 ambiguous bases ('N's), representing 23.47% of the total length. Despite this fragmentation, the assembly successfully recovered key photosynthetic genes and provided substantial genomic information from this less abundant template.

The annotation identified 84 different coding sequences (CDS) and 28 tRNA genes, including essential photosynthetic machinery such as photosystem I genes (psaB, psaC, psaJ), photosystem II genes (psbA, psbB, psbC, psbD, psbE, psbF, psbH, psbI, psbK, psbL, psbN), ATP synthase genes (atpA, atpB, atpE, atpH, atpI), and the large subunit of RuBisCO (rbcL). Additionally, NADH dehydrogenase genes (ndhA, ndhC, ndhD, ndhE, ndhG, ndhH, ndhI, ndhJ, ndhK) and several ribosomal protein genes were recovered.

**FIGURE 9:**
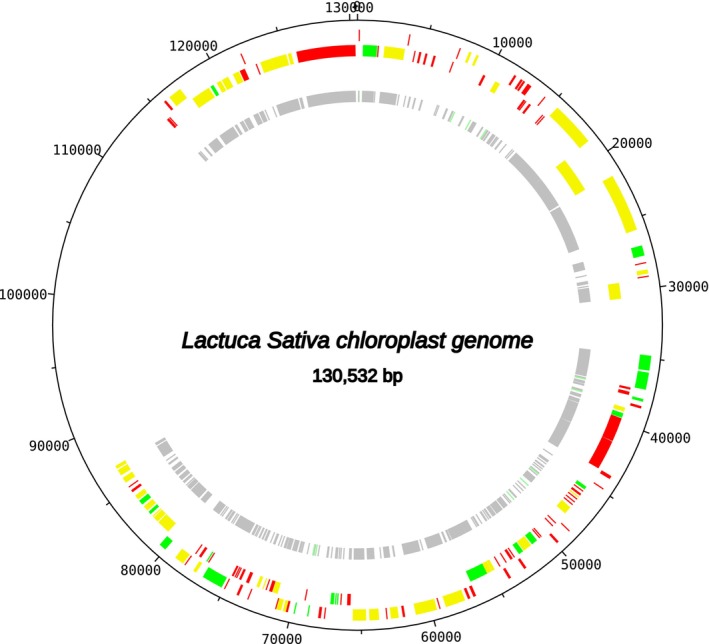
Schematic representation of the chloroplast genome of the *Lactuca sativa* assembled in this study.

However, the assembly lacked several critical components typically found in complete chloroplast genomes, including: (i) ribosomal RNA genes (rrn16, rrn23, rrn4.5, rrn5), which are essential for chloroplast protein synthesis; (ii) key photosystem I genes (psaA, psaI); (iii) important photosystem II genes (psbJ, psbM, psbT, psbZ); (iv) the atpF gene; (v) important NADH dehydrogenase genes (ndhB, ndhF); and (vi) conserved hypothetical chloroplast genes (ycf1, ycf2). The fragmented nature of this assembly reflects the low relative abundance of *L. sativa* DNA in the metagenomic sample compared to the dominant bacterial and mitochondrial templates (Figure 9 and Appendix C).

### 3.4 *Acetilactobacillus Jinshanensis* Strain AJITA Genome Assembly

To identify the most suitable reference for assembling the dominant bacterial genome, we employed a custom script (Species Match or SpM) to systematically rank candidate species based on their representativeness in the scaffold data. This script parses the tabular output (format 6) from the BLASTn search to calculate a score for each unique NCBI subject sequence. The process involves: (i) filtering for high‐identity alignments (≥ 99.8% identity) to focus on species‐level matches; (ii) calculating a weighted score for each alignment by multiplying its percent identity by its length; (iii) summing these weighted scores for each unique NCBI subject; and finally, (iv) normalizing each subject's total score against the sum of all weighted scores to yield a proportional representativeness. This score then ranked the subjects, and the top‐ranking hit, *Acetilactobacillus jinshanensis* (via its reference genome ASM435937v1), was selected as the reference for the subsequent assembly. This ranking logic was implemented using standard UNIX tools (awk, sort), ensuring the approach is reproducible.

**FIGURE 10:**
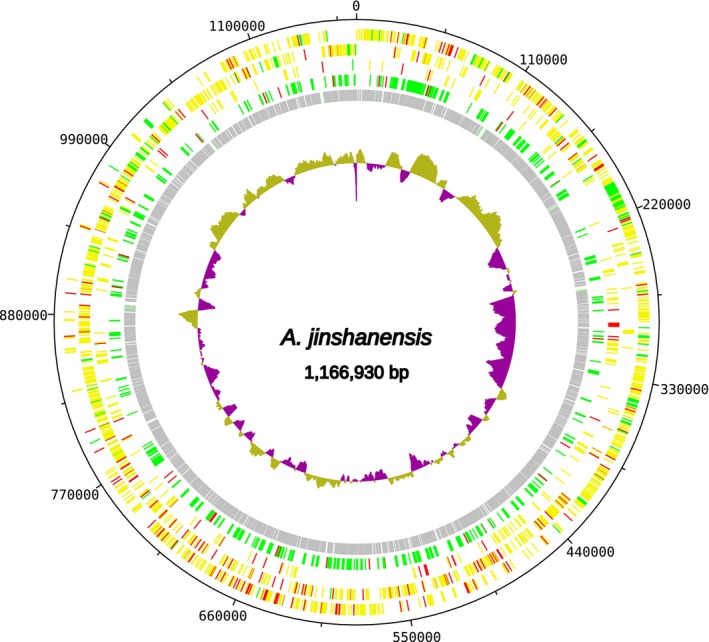
Schematic representation of the *Acetilactobacillus jinshanensis* strain ajita genome assembled in this study.

The assembled genome has a 41.45% GC content and spans 2,097,977 bp, containing 2094 coding sequences (CDS), four rRNA genes, and 81 tRNA genes. This assembly is highly comparable to the reference genome (ASM435937v1), which has a size of 2,097,928 bp and contains 2126 coding sequences. The assembly contains 19 gaps with a total of 50 ambiguous bases ('N's), representing 0.002% of the total genome length. Gap sizes ranged from 2 to 9 nucleotides (median: 2 nucleotides, mean: 2.6 nucleotides), indicating a high‐quality assembly with minimal unresolved regions. Despite these small gaps, the assembly successfully captured all essential bacterial genes and represents a near‐complete genome suitable for detailed genomic analyses (Figure 10). No antibiotic‐resistance genes were identified in the genome using ABRicate. Furthermore, no secondary metabolites were predicted using antiSMASH. This novel metagenome‐assembled genome (MAG) was deposited in the NCBI under the accession number CP171343.

To investigate the taxonomic status of the ajita strain, we performed whole‐genome comparisons against the *A. jinshanensis* type strain and other related species (Table 3). The Average Nucleotide Identity (ANI) between our assembly (CP171343.1) and the *A. jinshanensis* type strain (CP034726.1) ranged from 81.07% to 81.42% (FastANI, bidirectional), and between our assembly and another *A. jinshanensis* genome (CP187400.1) ranged from 81.20% to 81.39%. All values are well below the widely accepted 95%–96% threshold for species delineation. This finding was corroborated by digital DNA–DNA Hybridization (dDDH) analysis, which yielded values of 25%–27% between the genomes, far below the 70% species boundary. These results provide robust genomic evidence that strain ajita represents a distinct species from *A. jinshanensis*, constituting a putative novel species within the genus *Acetilactobacillus*.

## 4 Discussion

This study serves as a methodological proof of concept, demonstrating that deep shotgun sequencing of a single, complex environmental sample can yield a wealth of genomic resources and ecological insights across a wide range of template abundances. Among the various findings, genomic comparisons revealed evidence for a putative novel bacterial species. The differential assembly quality observed across the three recovered genomes—from the near‐complete bacterial chromosome to the fragmented chloroplast assembly—perfectly illustrates the method's capacity to recover genomic information across varying abundance levels while providing direct insight into the ecological composition of the sampled microenvironment.

The assembly quality gradient observed in this study, summarized in Table 2, represents a key methodological finding rather than a limitation. The near‐complete *Acetilactobacillus jinshanensis* genome (2,097,977 bp, 0.002% ambiguous bases in 19 small gaps) reflects the high abundance of this bacterial species in the larval food sample. In contrast, the draft‐quality mitochondrial genome of *T. angustula* (15,498–15,549 bp, core genes present but lacking rRNAs and a complete tRNA set) and the highly fragmented chloroplast genome of *L. sativa* (130,532 bp, 23.47% ambiguous bases, missing rRNAs and several key genes) demonstrate the method's sensitivity to template abundance while still recovering substantial genomic information from secondary components of the environmental DNA pool.

**TABLE 2:** Assembly quality gradient reflecting template abundance in the metagenomic sample.

Genome	Assembly status	Relative abundance	Size (bp)	*N* content (%)	Missing components
*A. jinshanensis*	Near‐complete	High	2,097,977	0.002	19 small gaps (2–9 bp)
*T. angustula* mito	Draft	Medium	15,498‐15,549	Not determined	rRNAs, nad3, nad4l, atp8, tRNAs
*L. sativa* chloro	Fragmented	Low	130,532	23.47	rRNAs, psaA/I, psbJ/M/T/Z, atpF, ndhB/F

**TABLE 3:** Genomic comparison of the *A. jinshanensis* strain ajita with its closest relatives.

Comparison pair	ANI (%)	dDDH (%)
*ajita* (this study) vs. *A. jinshanensis* (ref)	81.42	25.0
*ajita* (this study) vs. *A. jinshanensis* (CP187400.1)	81.39	25.5
*A. jinshanensis* (ref) vs. *A. jinshanensis* (CP187400.1)	99.99	—
*Species delineation thresholds: ANI* < 95%; *dDDH* < 70%		

This quality differential is not a methodological failure but rather an informative outcome that directly reflects the relative ecological importance of each organism within the sampled environment. Assembly completeness thus functions as a proxy for abundance, providing researchers with immediate insight into the dominant versus secondary components of complex environmental samples. The ability to simultaneously recover near‐complete genomes from abundant organisms alongside substantial partial assemblies from less abundant sources represents a significant advantage for biodiversity assessment and ecological characterization.

In this context, while 16S and ITS sequencing have provided valuable insights into the taxonomic composition of microbial communities associated with the larval food of stingless bees, the application of shotgun sequencing offers a more comprehensive and high‐resolution approach to microbial analysis. Additionally, we provided the first description of the microbial community associated with larval food for four species of Brazilian stingless bees (Santos et al. 2023), utilizing 16S and ITS sequencing.

Shotgun sequencing offers an extensive analysis by capturing all genetic material within a sample, facilitating a deep understanding of genomic content and biodiversity. This method contrasts sharply with 16S and ITS sequencing, which are targeted approaches focused on specific regions crucial for identifying and classifying microbial species (Klindworth et al. 2013; Abellan‐Schneyder et al. 2021). The widespread adoption of next‐generation sequencing has solved many earlier methodological limitations, allowing the routine sequencing of whole genomes (Cameron 2025). Shotgun sequencing enables the identification of microbes with greater precision, potentially down to the species or strain level, and provides valuable insights into the genetic makeup of a community (Pinto and Bhatt 2024). This makes it a powerful tool for exploring microbial diversity and environmental function.

In our previous work using 16S sequencing of the same sample of larval food of *T. angustula* used here, only three bacterial genera were identified, compared to 209 found here in an in‐depth shotgun analysis (Santos et al. 2023). The palynological analysis revealed 27 pollen types from 14 botanical families (Table 1), providing a comprehensive overview of the plant diversity accessed by the bees. Both technologies employ different approaches, and in‐depth shotgun sequencing is more effective at identifying less abundant taxa than 16S sequencing (Durazzi et al. 2021). Here, we used more than 50 GB of output. Shotgun sequencing offers several advantages over the 16S amplicon method, including improved detection of bacterial species, enhanced detection of diversity, and the ability to assemble genomes (Ranjan et al. 2016).

Only five genera of fungi were identified in this sample of *T. angustula* using shotgun sequencing. In previous work (Santos et al. 2023), the same sample identified 128 fungal genera (22 ASVs identified and 106 ASVs non‐identified) using ITS sequencing. Such comparative assessments between techniques face challenges, primarily with DNA extraction, for which a wide range of kits and protocols are designed to accommodate diverse sample types. A pivotal factor in comparing identical samples through 16S and ITS sequencing is the use of PCR to amplify targeted regions. This amplification can disproportionately enhance the DNA of predominant species within the sample, potentially masking the genetic material of less prevalent species (Schloss et al. 2011). However, our data showed that employing PCR to amplify the ITS region was considerably more effective for analyzing the fungal community in the larval food of *T. angustula* than shotgun sequencing, despite utilizing the same DNA extraction kit.

These findings highlight the intricate microbial interactions within stingless bee hives, where fermentation processes play a crucial role in the overall ecosystem. In contrast to honeybees, stingless bees deposit pollen in cerumen pots that allow for fermentation driven by microbial activity (Rosa et al. 2003). The diverse insect community identified in the larval food (Table D1) suggests complex ecological interactions within the hive environment, including potential contamination from external sources and symbiotic relationships. It is then used to produce larval food. Various bacterial species have been observed within stingless beehives, including fermentative, lactic acid‐producing, and non‐lactic acid‐producing bacteria. It has been identified that bacteria associated with these environments include genera *Lactobacillus*, *Leuconostoc*, *Enterococcus*, *Fructobacillus*, *Streptomyces*, and *Bacillus* (Rosa et al. 2003; Cambronero‐Heinrichs et al. 2019; Belina‐Aldemita et al. 2020; Santos et al. 2023). The yeast species *Starmerella meliponinorum* has been specifically isolated from *Tetragonisca angustula* in Brazil (Daniel et al. 2013).

It is essential to note that this study is based on a single sample collected from a single colony at a specific time and location. Therefore, the findings regarding taxonomic composition and diversity cannot be generalized to the entire *T. angustula* species, which is known to have a broad and variable diet depending on location and season. Instead, this work should be viewed as a high‐resolution snapshot that provides an unprecedented level of detail into the microcosm of a single hive, serving as a valuable proof of concept for the application of shotgun metagenomics in pollinator ecology. Shotgun sequencing facilitated the identification of 123 plant families within the larval food under analysis in this work. In traditional palynological analysis conducted by microscopy, although eight pollen types represented more than half the total diversity found, the remaining 19 appeared at least once in the counting method. The diversity observed was similar to that found in other studies using traditional palynological analysis (Ponciano and May 2021; Martins et al. 2023; dos Santos et al. 2024) and in another work of our group, a pollen sample collected from the same hive's pollen pots revealed only 12 distinct pollen types (Torres et al. 2018). In the Amazon, it has been documented that pollen storage from *T. angustula* can contain samples from 17 distinct botanical families (de Novais et al. 2015). Additionally, a recent review analyzing the floral diversity visited by *T. angustula* revealed that this bee visited plants from 88 different families, which reinforces their broad foraging range and significant role in pollination networks (Macêdo et al. 2023). These findings suggest that shotgun sequencing is a contemporary method that complements traditional palynological analysis in stingless bees. However, the high taxonomic diversity observed via shotgun sequencing, particularly the 123 plant families, must be interpreted with caution. While this sensitivity enables the detection of trace DNA from rare interactions, it is also prone to false positives resulting from the misannotation of short, fragmented contigs or highly conserved DNA regions shared across taxa. The traditional palynological analysis, which identified 14 families, likely represents the core, actively gathered food sources. At the same time, the metagenomic signal captures a broader, but potentially noisier, spectrum of environmental DNA present in the hive.

Employing complementary methodologies, such as shotgun next‐generation sequencing (NGS), to investigate the taxonomic composition of the larval food of *T. angustula* and other bee species is critical for addressing the current limited availability of genomic data related to Brazilian biodiversity (Hammer et al. 2023; Smutin et al. 2024; Caesar et al. 2025). This limitation is evident in the low proportion of scaffolds—only 12.61% of the dataset—that exhibit significant similarity to sequences available in the offline NCBI database (Da and Lira 2024; Zenker et al. 2024; Oliveira et al. 2024; Hausdorf 2025). This strikingly low match rate, found in just a single sample, is compelling evidence of the unique and potentially novel biodiversity present, not just within the *T. angustula* larval food microbiome and associated pollen/nectar sources, but likely reflecting broader gaps in genomic resources for Brazilian and Latin American ecosystems. It highlights an urgent need for targeted genomic and metagenomic sequencing initiatives focused on regional biodiversity to build more comprehensive reference databases, which are crucial for accurately assessing and understanding these rich environments (Vilaça et al. 2024).

Certain taxonomic identifications should be interpreted with caution. For instance, the genus *Habropoda*, a group of bees, has not yet been reported in Brazil (Packer 2023). In this study, we report only the best hit for each contig. Due to the presence of conserved DNA regions across genera or families and the limited representation of this taxon in the offline NCBI database, which is predominantly populated with sequences from North America and Europe, our analysis retrieved sequences from genera not currently documented in Brazil. Our findings underscore the need to expand genomic reference databases to more accurately represent the biodiversity of underrepresented regions, such as South America.

In this context, the environmental DNA (eDNA) has emerged as a transformative tool for investigating ecological relationships. Its ability to detect diverse organisms from minimal environmental samples provides a highly effective approach for biodiversity assessment, conservation efforts, and elucidating complex ecosystem interactions (Sahu et al. 2023). Integrating eDNA methodologies into ecological research significantly enhances our capacity to address pressing environmental challenges and deepen our understanding of ecosystem dynamics. The analysis of DNA from the larval food of *T. angustula* provides a valuable framework for investigating ecological interactions as eDNA. For instance, the detection of human and dog DNA (as indicated among the taxa identified in Figure 1) likely reflects the urban setting of the meliponary, situated near areas with high human and pet activity, where interactions with flowers visited by the bees, or proximity to the hive entrance, can lead to DNA deposition. Furthermore, this approach enables the identification of plants used as food sources, providing insights into direct interactions, such as competition among bees (most likely between *Frieseomellita* and *Bombus*) or other insects for resources (Bohmann et al. 2014; Thomsen and Sigsgaard 2019). It facilitates the detection of indirect interactions, through DNA left behind by insects or other animals visiting flowers or close to the hive (such as humans and pets), thereby expanding our understanding of trophic and ecological networks.

Beyond ecological interactions, genomic analyses of *T. angustula* provide fundamental insights into its genetic architecture and methodological considerations in genome assembly. The nuclear and mitochondrial genomes of *T. angustula* were recently sequenced, with sizes of 284.5 Mbp and 17,440 bp, respectively (Ferrari et al. 2024). In the present study, two assembly methodologies were employed, resulting in two *T. angustula* mitochondrial genome assemblies that were smaller than the previously published version. This discrepancy highlights the significance of methodological approaches in genomic assembly and their influence on the resulting genomic structure.

The sample was collected from an urban meliponary near an urban farm that cultivates various vegetables, including lettuce ( *Lactuca sativa* ). Integrating metagenomic analyses extends our understanding of how environmental factors influence biodiversity. The extensive genomic data in the offline NCBI database enabled the assembly of a chloroplast genome of *L. sativa* . This underscores the urban environment's influence on the sample's genomic composition, highlighting the potential of metagenomic analyses in such contexts.

Microbial communities also play a crucial role in shaping the biochemical landscape of stingless bee colonies. The bacterium *Lactobacillus jinshani* was originally isolated from a solid‐state vinegar culture used to produce Zhenjiang aromatic vinegar. Its genome was sequenced and published in 2020 (Yu et al. 2020).

An important additional finding of this study is the genomic evidence suggesting that the dominant bacterium in the larval food, strain ajita, represents a novel species. Both ANI (81.1%–81.4%) and dDDH (25%–27%) values against the type strain of *Acetilactobacillus jinshanensis* fall far below widely accepted species delineation thresholds (ANI < 95%–96%; dDDH < 70%). This discovery underscores the power of untargeted shotgun metagenomics not merely for profiling known diversity, but for genuine genomic discovery, even from a single sample. The presence of a novel, dominant bacterial species in the larval food of *T. angustula* opens new avenues for research into its specific role in fermentation, nutrient provision, and potential co‐evolution with its host.

Subsequently, this species was reclassified and renamed *Acetilactobacillus jinshanensis* (Zheng et al. 2020). The successful assembly of a near‐complete genome for the novel strain *Acetilactobacillus jinshanensis* strain ajita, directly from metagenomic data, represents a key resource generated by this study. This metagenome‐assembled genome (MAG) provides a permanent genetic resource for future functional and comparative genomic studies. Curiously, the smell of fermented pollen from *T. angustula* is slightly acetic. The microbiome present in the pollen of stingless bees facilitates the fermentation process, producing alcohol, which is subsequently converted into acetic acid (da Silva et al. 2024).

The methodological considerations revealed by this study provide important insights for future metagenomic approaches. The relationship between template abundance and assembly quality observed here establishes realistic expectations for the recovery of metagenomic genomes. Researchers should anticipate that dominant organisms will yield high‐quality, near‐complete assemblies suitable for detailed genomic analyses. At the same time, less abundant components will produce partial but still valuable assemblies sufficient for phylogenetic placement, functional gene analysis, and biodiversity assessment. This abundance–quality relationship is not a limitation but rather a feature that provides immediate ecological information about the sampled environment.

In conclusion, this case study demonstrates that a single deep shotgun sequencing run can serve as an efficient engine for generating multiple genomic resources of varying completeness levels and substantive biological insight. The quality gradient from near‐complete bacterial genomes to partial organellar assemblies from the same sample illustrates both the potential and realistic limitations of this approach. Additionally, our whole‐genome comparisons (ANI/dDDH) provided evidence that the dominant bacterium assembled here represents a putative novel species within the genus Acetilactobacillus. This method enables the simultaneous characterization of biodiversity, identification of food sources, and investigation of ecological interactions through an eDNA framework, with assembly quality serving as a direct indicator of ecological abundance. There is a pressing need to expand genomic datasets in public repositories with environmental samples from Brazil and across Latin America to enhance our understanding of regional biodiversity. Future research should build upon this proof‐of‐concept by expanding the sampling to different colonies, seasons, and locations to better understand the distinction between core and transient community members and to continue the crucial work of bioprospecting for novel genes and organisms in these unique neotropical ecosystems.

## Author Contributions

**Carlos Ueira‐Vieira:** conceptualization (equal), formal analysis (equal), funding acquisition (equal), project administration (equal), resources (equal), supervision (equal), visualization (equal), writing – original draft (equal), writing – review and editing (equal). **Ana Carolina Costa Santos:** conceptualization (equal), data curation (equal), formal analysis (equal), investigation (equal), methodology (equal), resources (equal), writing – review and editing (equal). **Thayane Nogueira Araújo:** investigation (equal), methodology (equal), writing – original draft (equal). **Solange Cristina Augusto:** conceptualization (equal), resources (equal), supervision (equal), writing – review and editing (equal). **Natanael Borges de Avila:** investigation (equal), methodology (equal). **Ana Maria Bonetti:** conceptualization (equal), funding acquisition (equal), resources (equal), supervision (equal), writing – original draft (equal). **Anderson Rodrigues dos Santos:** data curation (equal), formal analysis (equal), methodology (equal), software (equal), visualization (equal), writing – review and editing (equal).

## Funding

This work was supported by the Fundação de Amparo à Pesquisa do Estado de Minas Gerais (FAPEMIG—grant numbers APQ‐02766‐17, APQ‐00269‐22); Conselho Nacional de Desenvolvimento Científico e Tecnológico (CNPq—grant number 303667/2021‐4); and Coordenação de Aperfeiçoamento de Pessoal de Nível Superior—Brasil (CAPES—Finance Code 001).

## Conflicts of Interest

The authors declare no conflicts of interest.

## Data Availability

The data that support the findings of this study are openly available in GenBank at https://www.ncbi.nlm.nih.gov, reference number CP171343, PV951499, and PV951498.

## Appendix A

Bacterial genera identified in the metagenomic analysis.

**TABLE A1:** Complete list of bacterial genera identified in the larval food of *Tetragonisca angustula* through metagenomic shotgun sequencing.

#	Genus
1	*Acetilactobacillus*
2	*Acetobacter*
3	*Achromobacter*
4	*Acidocella*
5	*Acidovorax*
6	*Acinetobacter*
7	*Actinomadura*
8	*Actinosynnema*
9	*Aerococcus*
10	*Aggregatibacter*
11	*Agrobacterium*
12	*Algoriphagus*
13	*Alicyclobacillus*
14	*Allofrancisella*
15	*Allopseudocardia*
16	*Anaerosporobacter*
17	*Anoxybacillus*
18	*Apilactobacillus*
19	*Aquaricoccus*
20	*Arthrobacter*
21	*Asticcacaulis*
22	*Atopobium*
23	*Azorhizobium*
24	*Azotobacter*
25	*Bacillus*
26	*Bacteroidetes*
27	*Bacteroides*
28	*Bdellovibrio*
29	*Bifidobacterium*
30	*Bordetella*
31	*Borrelia*
32	*Bradyrhizobium*
33	*Brucella*
34	*Burkholderia*
35	*Buttiauxella*
36	*Caldibacillus*
37	*Candidatus*
38	*Caulobacter*
39	*Cellulophaga*
40	*Cereibacter*
41	*Chitinophaga*
42	*Chryseobacterium*
43	*Citrobacter*
44	*Clostridium*
45	*Comamonas*
46	*Companilactobacillus*
47	*Corynebacterium*
48	*Cronobacter*
49	*Curtobacterium*
50	*Curvibacter*
51	*Dactylosporangium*
52	*Delftia*
53	*Desulfuromonas*
54	*Devosia*
55	*Dyella*
56	*Enterobacter*
57	*Enterococcus*
58	*Erwinia*
59	*Escherichia*
60	*Exiguobacterium*
61	*Flavobacterium*
62	*Francisella*
63	*Frankia*
64	*Fructilactobacillus*
65	*Geobacillus*
66	*Gilliamella*
67	*Gluconobacter*
68	*Gordonia*
69	*Halobacillus*
70	*Hansschlegelia*
71	*Herbaspirillum*
72	*Hydrogenophilus*
73	*Hymenobacter*
74	*Isoptericola*
75	*Janthinobacterium*
76	*Kitasatospora*
77	*Klebsiella*
78	*Kosakonia*
79	*Lactiplantibacillus*
80	*Lactobacillus*
81	*Lactococcus*
82	*Leclercia*
83	*Leifsonia*
84	*Lentilactobacillus*
85	*Leuconostoc*
86	*Levilactobacillus*
87	*Ligilactobacillus*
88	*Limosilactobacillus*
89	*Liquorilactobacillus*
90	*Listeria*
91	*Lysinibacillus*
92	*Magnetospirillum*
93	*Massilia*
94	*Mesorhizobium*
95	*Methylobacterium*
96	*Microbacterium*
97	*Micrococcus*
98	*Micromonospora*
99	*Mucilaginibacter*
100	*Mycobacterium*
101	*Mycobacteroides*
102	*Mycoplasma*
103	*Neisseria*
104	*Nicoliella*
105	*Nitrosomonas*
106	*Nocardia*
107	*Nocardioides*
108	*Oenococcus*
109	*Ornithinimicrobium*
110	*Paenibacillus*
111	*Pantoea*
112	*Paraburkholderia*
113	*Paracoccus*
114	*Pasteurella*
115	*Pediococcus*
116	*Pelobacter*
117	*Photobacterium*
118	*Photorhabdus*
119	*Phyllobacterium*
120	*Planococcus*
121	*Planomicrobium*
122	*Polaromonas*
123	*Polynucleobacter*
124	*Pseudogulbenkiania*
125	*Pseudomonas*
126	*Providencia*
127	*Psychrobacter*
128	*Ralstonia*
129	*Ramlibacter*
130	*Raoultella*
131	*Rhizobium*
132	*Rhodanobacter*
133	*Rhodococcus*
134	*Rhodopseudomonas*
135	*Rhodospirillum*
136	*Riemerella*
137	*Roseomonas*
138	*Salmonella*
139	*Scardovia*
140	*Serratia*
141	*Shewanella*
142	*Shigella*
143	*Sinorhizobium*
144	*Sphingobium*
145	*Sphingomonas*
146	*Spiroplasma*
147	*Sporosarcina*
148	*Staphylococcus*
149	*Stenotrophomonas*
150	*Streptococcus*
151	*Streptomyces*
152	*Synechococcus*
153	*Tatumella*
154	*Thermomonospora*
155	*Thiobacillus*
156	*Thiomonas*
157	*Treponema*
158	*Tumebacillus*
159	*Unclassified*
160	*Vagococcus*
161	*Variibacter*
162	*Variovorax*
163	*Vibrio*
164	*Weissella*
165	*Wolbachia*
166	*Xanthomonas*
167	*Xylella*
168	*Yersinia*
169	*Zymomonas*
170	*Zymobacter*

## Appendix B

Fungal genera identified in the metagenomic analysis.

**TABLE B1:** Complete list of fungal genera identified in the larval food of *Tetragonisca angustula* through metagenomic shotgun sequencing.

#	Genus
1	*Aspergillus*
2	*Blumeria*
3	*Erysiphe*
4	*Sclerotinia*
5	*Talaromyces*

## Appendix C

Plant families and genera identified in the metagenomic analysis.

**TABLE C1:** Complete list of plant families and genera identified in the larval food of *Tetragonisca angustula* through metagenomic shotgun sequencing.

#	Family	Genus	Species
1		*Ranunculaceae*	
2	Acanthaceae	*Avicennia*	
3	Achariaceae	*Erythrospermum*	
4	Actinidiaceae		
5	Adoxaceae		
6	Amaranthaceae		
7	Anacardiaceae	*Mangifera*	
8	Apiaceae		
9	Apiaceae		
10	Apiaceae		
11	Apiaceae		
12	Apiaceae		
13	Apiaceae		
14	Apiaceae		
15	Apiaceae		
16	Apiaceae		
17	Apiaceae		
18	Apiaceae		
19	Apiaceae		
20	Apiaceae		
21	Apiaceae		
22	Apiaceae		
23	Apiaceae		
24	Apiaceae		
25	Apiaceae		
26	Apiaceae		
27	Apiaceae		
28	Apiaceae		
29	Apiaceae		
30	Apiaceae		
31	Apiaceae		
32	Apiaceae		
33	Apiaceae		
34	Apiaceae		
35	Apiaceae		
36	Apiaceae		
37	Apiaceae		
38	Apiaceae		
39	Apiaceae		
40	Apiaceae		
41	Apiaceae		
42	Apiaceae		
43	Apiaceae		
44	Apiaceae		
45	Apiaceae		
46	Apiaceae		
47	Apiaceae		
48	Apiaceae		
49	Apiaceae		
50	Apiaceae		
51	Apiaceae		
52	Apiaceae		
53	Apiaceae		
54	Apiaceae		
55	Apiaceae		
56	Apiaceae		
57	Arecaceae	*Cocos*	
58	Arecaceae	*Phoenix*	
59	Arecaceae	*Phoenix*	
60	Arecaceae	*Phoenix*	
61	Arecaceae	*Phoenix*	
62	Arecaceae	*Phoenix*	
63	Arecaceae	*Phoenix*	
64	Asteraceae		
65	Asteraceae		
66	Asteraceae		
67	Asteraceae		
68	Brassicaceae		
69	Chenopodiaceae	*Chenopodium*	
70	Convolvulaceae	*Ipomoeeae*	
71	Elaeocarpaceae		
72	Ericaceae		
73	Euphorbiaceae		
74	Euphorbiaceae		
75	Fabaceae	*Arachis*	
76	Fabaceae	*Lotus*	
77	Fabaceae	*Vigna*	
78	Gesneriaceae	*Haberlea*	
79	Malvaceae		
80	Malvaceae	*Malvoideae*	
81	Musaceae	*Musa*	
82	Musaceae	*Musa*	*Musa acuminata*
83	Musaceae	*Musa*	
84	Musaceae	*Musa*	
85	Oleaceae	*Oleeae*	
86	Passifloraceae	*Passiflora*	
87	Poaceae	*Cenchrus*	
88	Polygonaceae	*Polygonum*	
89	Rosaceae		
90	Rubiaceae	*Coffea*	
91	Rubiaceae	*Coffea*	
92	Rubiaceae	*Mitriostigma*	
93	Rubiaceae		
94	Rubiaceae		
95	Simaroubaceae		
96	Solanaceae		

## Appendix D

Insect taxa identified in the metagenomic analysis.

**TABLE D1:** Complete list of insect taxa identified in the larval food of *Tetragonisca angustula* through metagenomic shotgun sequencing.

#	Family	Genus	Species
1	Acrididae	*Schistocerca*	
2	Andrenidae	*Andrena*	
3	Andrenidae	*Hoplandrena*	
4	Andrenidae	*Taeniandrena*	
5	Apidae	*Apis*	
6	Apidae	*Bombus*	
7	Apidae	*Frieseomelitta*	
8	Apidae	*Nomada*	
9	Apidae	*Tetragonisca*	
10	Araneidae		
11	Araneidae	*Meta*	
12	Araneidae	*Metellina*	
13	Culicidae	*Anopheles*	
14	Curculionidae	*Apoderus*	
15	Halictidae	*Lasioglossum*	
16	Halictidae	*Seladonia*	
17	Megachilidae	*Megachile*	
18	Tortricidae	*Apotomis*	
19	Sesiidae	*Bembecia*	
20	Andrenidae	*Callandrena*	
21	Lepidoptera		
22	Coleoptera		
23	Diptera		
24	Hymenoptera		
